# Targeted Drug Delivery for the Treatment of Blood Cancers

**DOI:** 10.3390/molecules27041310

**Published:** 2022-02-15

**Authors:** Yao Jiang, Weifeng Lin, Linyi Zhu

**Affiliations:** 1 Nuffield Division of Clinical Laboratory Sciences, Radcliffe Department of Medicine, University of Oxford, John Radcliffe Hospital, Oxford OX3 9DU, UK; yao.jiang@ndcls.ox.ac.uk; 2 Institute of Clinical Sciences, University of Birmingham, Birmingham B15 2TT, UK; 3 Department of Molecular Chemistry and Materials Science, Weizmann Institute of Sciences, Rehovot 761001, Israel; lin.weifeng@weizmann.ac.il; 4 Arthritis Research UK Centre for Osteoarthritis Pathogenesis, Kennedy Institute of Rheumatology, University of Oxford, Oxford OX3 7FY, UK

**Keywords:** blood cancers, drug delivery, nanomedicines

## Abstract

Blood cancers are a type of liquid tumor which means cancer is present in the body fluid. Multiple myeloma, leukemia, and lymphoma are the three common types of blood cancers. Chemotherapy is the major therapy of blood cancers by systemic administration of anticancer agents into the blood. However, a high incidence of relapse often happens, due to the low efficiency of the anticancer agents that accumulate in the tumor site, and therefore lead to a low survival rate of patients. This indicates an urgent need for a targeted drug delivery system to improve the safety and efficacy of therapeutics for blood cancers. In this review, we describe the current targeting strategies for blood cancers and recently investigated and approved drug delivery system formulations for blood cancers. In addition, we also discuss current challenges in the application of drug delivery systems for treating blood cancers.

## 1. Introduction

Cancers are one of the leading causes of death in the world [1]. Unlike solid tumors such as those in organs, blood cancers (including multiple myeloma, leukemia, and lymphoma) form in the bone marrow or in the lymphatic system [2,3]. Table 1 provides an overview of different types of blood cancers. Current treatments for blood cancers consist of chemotherapy, radiotherapy, immunotherapy, and transplantation [4]. Although, many chemotherapeutic drugs are clinically available for the treatment of blood cancers, there are no curative treatment approaches in clinical practice for these types of cancers due to the inevitable aggravation of blood cancers and bone metastasis [5]. Furthermore, it is difficult to achieve a sufficient therapeutic dose of anticancer agents at tumor sites inside bone marrow or the lymphatic system to suppress tumor growth via systematic administration [6]. To maintain therapeutic levels in bone marrow or the lymphatic system, chemotherapeutics require high dosage and/or more frequent administration which can result in increased side effects [7]. In addition, the bone marrow microenvironment contains a huge number of hematopoietic stem/progenitor cells which are resistance to chemotherapy and mediate disease refractory/relapse [5]. Therefore, a targeted drug delivery system for blood cancers is a significant challenge for chemotherapy.

In recent years, different types of nanoparticles have received considerable attention for the treatment of various types of solid tumors, leading to several successful drug delivery systems that have entered clinical practice. However, most of them have been done in solid tumors and few works have been done to develop drug delivery systems for the treatment of blood cancers. In this review, we summarized currently available strategies for drug delivery systems for treating blood cancers.

## 2. Targeting Delivery Strategy

### 2.1. Targeting Bone Marrow and Its Microenvironment

The bone marrow microenvironment plays a critical role in the maintenance of cell renewal and differentiation, especially for cancer cells. The bone marrow contains numerous blood vessels and capillaries. It is considered to be one of the most complex systems comprising various cell types including endothelia cells, stromal cells, osteocytes, fibroblasts, mesenchymal stem cells, macrophages, osteoclasts, and osteoblasts. Moreover, the non-cellular component including the extracellular matrix, oxygen tension, cytokines, and mechanical forces are also essential for cancer cell proliferation and are related to resistance. Targeting the bone marrow microenvironment can be improved by drug delivery systems and can be achieved passively or actively.

#### 2.1.1. Passive Targeting Strategy

Potentially beneficial properties of nanotherapeutics include improved bioavailability, reduced toxicity, greater dose response, and enhanced solubility as compared with conventional medicines [8]. Passive targeting depends on accumulation of the drug delivery system at a specific end organ or tumor site, through leaky vasculatures which mainly require a delivery system for its own characteristics including the size, shape, surface zeta-potential, and other properties. In the bone marrow, the drug accumulation amounts in the bone are related to the reticulo-endothelial cells in vessels [9]. The enhanced permeability and retention (EPR) effect in solid tumors was described more than 30 years ago, however, it has been less appreciated in blood cancers in contrast to solid tumors [10]. Particle size plays a critical role since the transcellular route takes place through the fenestrae between the endothelial cells in the bone marrow [11]. It has been reported that the sizes of the fenestrae in the endothelial wall are less than 150 nm which means the particles larger than 150 nm would be less likely to pass through [12]. Furthermore, nanoparticles smaller than 60 nm can penetrate and distribute into the bone marrow interstitial space, since reticuloendothelial sinusoidal blood capillaries consist of pores as large as 60 nm in diameter [13]. In addition, to achieve high efficiency for drug delivery in bone marrow, a long circulation time in blood vessels is critical for nanomedicine. Liposomes less than 100 nm in diameter circulate longer in the blood and have less interaction with plasma proteins. However, there is also a limitation of nanoparticles with a small size, since nanoparticles less than 50 nm limit the drug encapsulation efficiency [14]. In addition to the role of diameter size in nanomedicine, surface charge also plays a major role in bone marrow uptake. It has been reported that negatively charged liposomes increased the efficiency of bone marrow uptake rate by the macrophages [11]. Therefore, the ideal size of nanoparticles for blood cancers should be between 50 nm and 100 nm.

#### 2.1.2. Targeting Bone Surface-Mediated Bone Marrow

**Figure 1:**
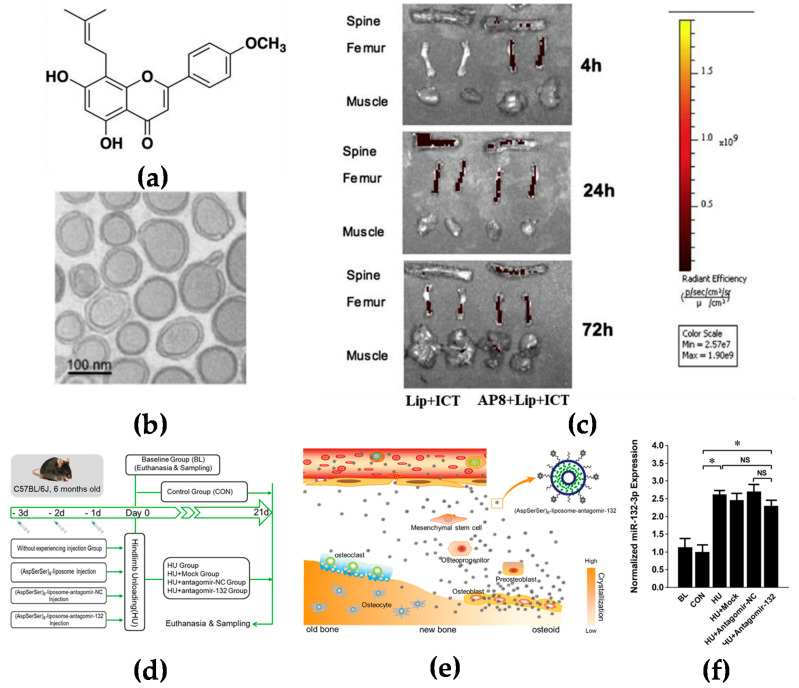
Targeting the bone surface-mediated bone marrow: (**a**) Characterization of the bone-targeting delivery system in vitro and in vivo. Chemical structure of icaritin (ICT); (**b**) the morphology of Asp8-liposome-icaritin taken by cryo-transmission electron microscope (Cryo-TEM) (scale bar = 100 nm, magnification = 10^5^×); (**c**) localization of fluorescent-labeled liposome delivery system with or without Asp8 targeting peptide in mice by optical imaging IVIS analysis, 72 h after administration; (**d**) a schematic diagram to illustrate the experimental design for targeted delivery of antagomir-132 to specifically decrease miRNA-132-3p levels in bone; (**e**) a schematic diagram to illustrate how antagomir-132 is selectively delivered to bone formation region by (AspSerSer)_6_; (**f**) analysis of miRNA-132-3p expression in the femur bone tissues of mice after hindlimb unloading for 21 days. BL—baseline group, mice were euthanatized and sampled at the beginning of experiment; CON—control group mice were raised in normal conditions during the experiment; HU—hindlimb unloading group, mice were submitted to a hindlimb unloading experiment; HU + Mock—hindlimb unloading plus (AspSerSer)_6_-liposome injection group, mice were injected with the (AspSerSer)_6_-liposome before HU; HU + antagomir-NC—hindlimb unloading plus (AspSerSer)_6_-liposome-antagomir-NC injection group, mice were injected with the (AspSerSer)_6_-liposome-antagomir-NC before HU; HU + antagomir-132—hindlimb unloading plus (AspSerSer)_6_-liposome-antagomir-132 injection group, mice were injected with the (AspSerSer)_6_-liposome-antagomir-132 before HU. Values are shown as mean ± SD, *n*  =  6. * *p*  <  0.05. NS, not significant. (**a**–**c**) Adapted with permission from [17] and (**d**–**f**) adapted with permission from [19].

Bone is rich in hydroxyapatite which has a high affinity with glutamic acid or aspartic acid [15]. It has been reported that several oligopeptides have demonstrated their particular interactions with bone tissues. Eight repetitive aspartic acids, also known as Asp8, is one of the successful examples which has been reported to bind to bone-resorption surfaces [16]. Asp8 has been demonstrated to mainly bind to the highly crystallized hydroxyapatite of bone-resorption surfaces. It was found that Asp8-icaritin-liposome enhanced bone formation in ovariectomized mice as compared with an icaritin-liposome control lacking the Asp8 moiety (Figure 1a–c) [17]. (AspSerSer)_6_ is another type of oligopeptide used for the bone-surface delivery that mainly binds to calcium phosphate which is mainly distributed in the mantle dentin in the bone [18]. Hu et al. used the (AspSerSer)_6_-cationic liposome system to deliver miRNA-132-3p in bone, resulting in prevention and treatment of osteoporosis (Figure 1d–f) [19].

Another type of stable bone targeting by high affinity to hydroxyapatite is bisphosphonates [16]. Bisphosphonates as radionuclides have been reported for delivery of antineoplastic compounds into the bone marrow [20]. Furthermore, some studies have also demonstrated that bisphosphonates can reduce bone metastasis by preventing osteoclast differentiation [21]. Tian et al. reported that antibody conjugated bisphosphonates could deliver therapeutic antibodies directly into the bone for treatment of bone metastatic cancers and other bone diseases [22]. Therefore, bone-targeting molecules that are capable of binding to bone-formation surfaces may precisely deliver therapeutic agents to the bone marrow niche for blood cancer therapy.

#### 2.1.3. Active Targeting

**Figure 2:**
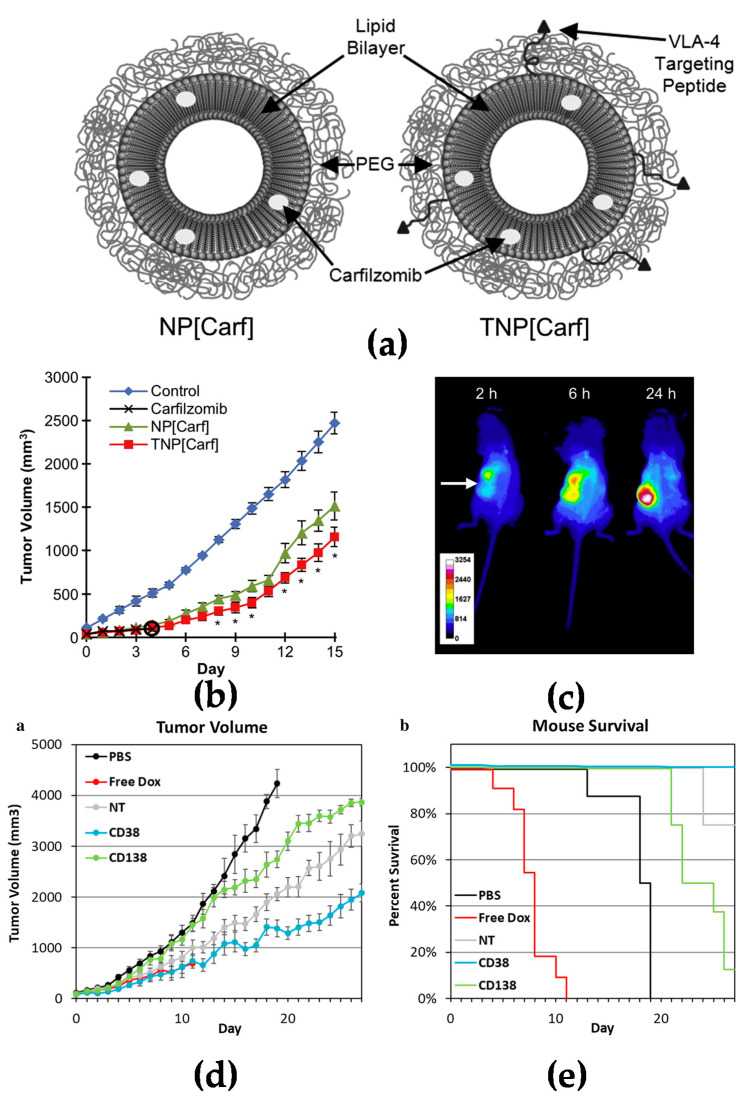
Active targeting strategy by peptide or antibody mediated nanomedicines: (**a**) Illustration of PEGylated non-targeted liposomal carfilzomib nanoparticles (NP[Carf], left) and VLA-4 targeted liposomal carfilzomib nanoparticles (TNP[Carf], right); (**b**) liposomal carfilzomib nanoparticles preferentially accumulate in the tumor, inhibit tumor growth, and reduce systemic toxicities in vivo. Tumor bearing SCID mice were injected intravenously on Days 1, 2, 8, and 9 with NP[Carf], TNP[Carf], free carfilzomib, and PBS at a dose of 5 mg/kg carfilzomib equivalence. Tumor growth inhibition was measured via calipers; (**c**) in vivo images of near infrared dye loaded targeted nanoparticles in tumor bearing mice. Images were taken for all mice at t = 2, 6, and 24 h using non-invasive methods. The representative images show the accumulation of the nanoparticles in the tumor (white arrow) over time; (**d**,**e**) in vivo efficacy of CD38pep- and CD138pep-targeted nanoparticles loaded with prodrug doxorubicin. Nanoparticles targeted with CD38pep or CD138pep were prepared loaded with a doxorubicin prodrug and their in vivo efficacy was tested against that of free doxorubicin in a subcutaneous xenograft mouse model. Mice were injected with H929 cells and tumors were allowed to grow to a predetermined size before i.v. injection of nanoparticle formulations began on Day 1. Mice were injected with 3 mg/kg of doxorubicin or nanoparticle prodrug equivalent on Days 1, 3, 5, 7, and 9. Tumor volume (**d**) and survival (**e**) were tracked with mice being killed when tumor volume grew too large or mouse weight was too low. *n* = 6 for all groups and data represent means (± s.e.m.). (**a**–**c**) Adapted with permission from [26] and (**d**–**e**) adapted with permission from [28]).

Active targeting increases specific delivery to tumor tissues. Many blood cancer cells express specific surface biomarkers which can be specifically targeted by coupling peptides/antibodies/ligands to the surface of nanomedicines for drug delivery [23,24]. Targeting nanomedicines modified with peptides is a common strategy investigated extensively in drug delivery research. Liposomes modified with RGD peptide (Arg-Gly-Asp) have been widely used to target angiogenic endothelial cells in tumors [25]. It has also been reported that liposomes conjugated with a cyclic pentamer peptide (VLA-4, very late antigen-4) can be used to target multiple myeloma (Figure 2a–c) [26]. Targeting via antibodies is another approach for an active targeting strategy [27]. It is known that CD38 and CD138 are widely expressed on multiple myeloma cells. Liposomes modified with anti-CD38 or anti-CD138 monoclonal antibody could be a new approach for a targeted delivery system with both targeting myeloma cells and also delivery anticancer agents to cancer cells (Figure 2d,e) [28]. Similar to CD38 and CD138, CD19 is one of the markers expressed in most of the lymphoma diseases [29]; an anti-CD19 targeted liposome encapsuled rapamycin showed promising lymphoma cell-specific treatment inducing autophagic cell death [30,31].

### 2.2. Targeting Spleen and Lymphoid Nodes

Spleen and lymph nodes provide a distinct microenvironment for tumor cells in blood cancers. The spleen is considered to be involved in many blood cancers, especially in lymphomas. It has been reported that the spleen also plays a key role in tumor immunity by recruiting monocytes and macrophages to the tumor tissues [32]. Spleen involvement is found in approximately one third of lymphomas and can also upstage the disease, especially in Hodgkin lymphoma [33]. Intravenously administered nanoparticles tend to target the spleen because of the phagocytic activity of monocytes and macrophages [34]. In vivo experiments have shown that siRNA encapsuled nanoparticles can reduce tumor growth [35]. Enhanced drug concentration in the spleen has also provided therapeutic benefits in spleen resident infections and hematological disorders including malaria, hairy cell leukemia, idiopathic thrombocytopenic purpura, and autoimmune hemolytic anemia [36].

Lymph nodes initiate most immune responses which can prevent malignant transformation [37]. Antitumor immune responses are still active in some malignancies, impacting progression and outcome. In addition, the cytokines in lymphoid nodes also provide a proinflammatory microenvironment which can also support proliferation of malignant cells [38].

### 2.3. Targeting Vascular System

Neovascularization is always associated with poor prognosis in most blood cancers including acute myeloid leukemia, multiple myeloma, acute lymphatic leukemia, chronic lymphatic leukemia, and Burkett’s lymphoma [39]. Endothelial surface receptors are highly expressed on the inner lining of blood vessels. Shamay et al. reported that vascular endothelial growth factor receptor 1 (VEGFR1)-targeted polymer drug conjugates showed efficient antitumor effect by targeting tumor vasculature [40]. Another strategy is to utilize tumor-homing immunocytokines such as interleukin-2 (IL-2) [41]. The antibody-based delivery of IL-2 to extracellular targets expressed in the easily accessible tumor-associated vasculature showed therapeutic potential for acute myeloid leukemia and other solid tumors [42]. E-selectin is mainly expressed on inflamed endothelial cells which always exist in the vasculature of inflammatory and tumor sites [43]. Gholizadeh et al. reported that E-selectin targeted immunoliposomes could delivery rapamycin, which specifically inhibited inflammatory responses in inflamed endothelial cells [44]. Targeting the vascular system can direct antiangiogenic agents to the blood vessels to suppress angiogenesis, and can also contribute released chemotherapeutic drugs to inhibit cell proliferation near the vascular in the bone marrow. A vascular targeting co-delivery strategy can maximize the combination therapeutic efficacy for the treatment of blood cancers.

## 3. Nanomedicines for Blood Cancers

### 3.1. Multiple Myeloma

Multiple myeloma (MM) is a B cell malignancy disease which is characterized by the accumulation of malignant plasma cells in the bone marrow. Although the new treatment and transplant has been utilized in recent decades and has prolonged the overall survival for patients, multiple myeloma is still not curable since it is difficult to remove the tumor cells from the bone marrow. Swami et al. reported that PEG-PLGA encapsuled bortezomib nanoparticles inhibited myeloma growth in a mouse model [5]. Ashley et al. reported that carfilzomib-loaded liposomal nanoparticles targeted myeloma cells [26]. A doxorubicin liposome combined with bortezomib for the treatment of relapsed or refractory multiple myeloma has already been approved by FDA for clinical use [45]. The outcome was based on a phase III clinical trial which showed that liposomal doxorubicin was superior to bortezomib monotherapy [46].

In recent years, protease inhibitors have been widely used in the treatment of multiple myeloma [47]. Nanoparticles encapsuled with protease inhibitors have also been investigated. Lee et al. reported on an injectable nanomedicine for MM therapy by encapsulating bortezomib (class I protease inhibitor) in nanoparticles that possessed a catechol-functionalized polycarbonate core through a pH-sensitive covalent bond between the biodegradable phenylboronic acid in bortezomib and catechol [48]. An in vitro release study showed that, at pH 7.4, the bortezomib release from the composite remained low at 7%, whereas in an acidic environment, ∼85% of bortezomib was released gradually over 9 days. In vivo studies showed that tumor progression of mice treated with the bortezomib-loaded micelle/hydrogel composite resulted in significant delay in tumor progression in a xenograft mouse model, thus, demonstrating the potential of the hydrogel for subcutaneous administration and sustained drug delivery. In addition, an antibody-based delivery system has also been investigated in the myeloma treatment. Huang et al. reported the development of monoclonal anti-CD38 antibody conjugated nanoparticles encapsulated with S3I-1757 (a STAT3 inhibitor) in MM therapy [49]. In this study, they generated two nanoparticle delivery systems with or without the anti-CD38 antibody. The in vitro release study showed two formulations with a comparable drug release (about 68%) property. However, the anti-CD38 antibody coated nanoparticles showed increased drug uptake in two different MM cell lines. In vivo studies performed on a xenograft mouse model demonstrated that the anti-CD38 antibody coated nanoparticles were able to significantly reduce tumor size by four-fold as compared with non-coated nanoparticles, after 12 days drug administration, which indicated that the anti-CD38 antibody on nanoparticles loaded with STAT3 inhibitors can further improve their therapeutic effects against MM.

### 3.2. Acute Myeloid Leukemia

Acute myeloid leukemia (AML) is another common type of hematological malignancy which is characterized by high proliferation of abnormal myeloblasts in the bone marrow [50,51]. Chemotherapy is still the primary choice for AML treatment. However, the overall survival of single chemotherapy for AML patients is still very low [52]. A combination of two or more anticancer reagents is often used for AML therapy to increase the treatment outcome, but various adverse effects can happen during the treatments [53]. Recently, there are some drug delivery systems that have been investigated to increase the anti-AML effect. Roboz et al. reported on a lipid-drug conjugate encapsuled cytarabine that has been put into a phase III clinical trial [54]. Alakhova et al. reported on a pluronic-based micelle which could increase the anti-AML efficacy of doxorubicin and was also in a phase III clinical trial [55]. Tardi et al. reported on a cytarabine liposome which could increase accumulation in leukemia cells inside the bone marrow and enhance efficacy in AML xenograft model [56].

In 2017, CPX-351 (trade name Vyxeos) was approved by the FDA and EMA for treating newly diagnosed therapy-related AML and/or AML with myelodysplasia-related changes [57]. CPX-351 was initially synthesized and evaluated in in vitro and in vivo studies with leukemia cell lines. The results indicated that the liposomal-encapsulated cytarabine and daunorubicin could display the best synergistic effect and minimum antagonism at a ratio of 5:1, with higher proportions of response rates, more durable remissions, and longer maintenances in bone marrows as compared with a free drug cocktail of cytarabine and daunorubicin with their maximum tolerated doses. Lancet et al. analyzed the data from the clinical trials and found that CPX-351 indicated a significant improvement in survival over standard induction chemotherapy for high-risk patients with AML, older patients with sAML, and a poor-risk subgroup of patients with AML [58].

AZD2811 polymeric nanoparticles are loaded with aurora kinase B inhibitor. AZD2811 has been assessed in AML xenograft models and has shown improved efficacy in inhibiting tumor growth and inducing apoptosis as compared with free aurora kinase B inhibitor (AZD1152). Moreover, this formulation has also demonstrated transient cellular reduction in bone marrow, and may be a potential agent for targeting residual disease. There are two clinical trials ongoing for evaluating the safety, tolerability, and pharmacokinetics of AZD2811 (NCT02579226, NCT03217838) [59].

### 3.3. B Cell Lymphomas

Lymphoma is a type of cancer which often happens in lymph nodes. The majority arise from B cells, and therefore, are called B cell lymphomas which include both Hodgkin’s lymphomas and most non-Hodgkin lymphomas [60]. Chemotherapy and stem cell transplantation are two main treatments for B cell lymphomas; however, relapse is often inevitable [61]. Antibody conjugates provided a new way for targeting therapy for B cell lymphomas. Brentuximab vedotin (Adcetris^®^, Seattle Genetics, Bothell, WA, USA) and ibritumomab tiuxetan (Zevalin^®^, IDEC, Cambridge, MA /Spectrum, Irvine, CA, USA) are two commercially available antibody-drug conjugates for Hodgkin lymphoma and non-Hodgkin lymphoma which have already been approved by the FDA [62,63]. Furthermore, new technology provides the possibility to selectively deliver anticancer agents to malignant cells without damaging healthy cells or systemic toxicity, allowing them to reach the lymph nodes. Nevala et al. reported on a nano-antibody targeted chemotherapy delivery system that used a slight modification of existing cancer drugs with significantly improved treatment efficacy in CD20+ B-cell lymphoma [64]. Martucci et al. reported on siRNA targeting Bcl2-loaded diatomite nanoparticles that demonstrated significant biological improvements for personalized treatment of lymphomas [65]. Choi et al. reported on dually targeted siRNA nanoformulation constructed using layer-by-layer nanoparticles (LbL-NP) for the treatment of non-Hodgkin lymphoma. The LbL-NP protects siRNA from nucleases in the bloodstream by embedding within polyelectrolyte layers that coat a polymeric core. The outermost layer consists of hyaluronic acid (a CD44 ligand) covalently conjugated to CD20 antibodies. The CD20/CD44 dual-targeting outer layer provides precise binding to blood cancer cells, followed by receptor-mediated endocytosis of the LbL-NP. The dual-targeting approach significantly enhances internalization of BCL-2 siRNA in lymphoma and leukemia cells, which leads to significant downregulation of BCL-2 expression. An in vivo study has demonstrated that systemic administration of the dual-targeted, siRNA-loaded nanoparticles induced apoptosis and hampered proliferation of blood cancer cells, both in cell culture and in orthotopic non-Hodgkin’s lymphoma animal models [66].

## 4. Challenges in Drug Delivery Systems for Treating Blood Cancers

To date, only a few targeted nano-based drug delivery systems are available in clinical practice. There are still many challenges and issues that need to be resolved. Although nano-based medicines provide a significant advantage in delivery strategies, it is still difficult to develop successful formulations that can be put into clinical use. There are biological challenges and non-biological challenges which are described below.

### 4.1. Biological Challenges

#### 4.1.1. Characterization of Nano-Based Medicines

One of the key points in the development of nano-based medicines is the stability and the efficiency of the loading drugs inside the drug delivery systems. In the design of drug delivery systems such as nanoparticles or liposomes, we can tune the polymer/lipid properties and introduce specific side groups, for instance, to increase the compatibility between the materials and drugs to be loaded. It is possible in the lab to change the properties of a nanocarrier such as molecular weight, ratio of hydrophobic/hydrophilic block, and concentration of drug carrier in relation to the drug to optimize the best property (the most anti-tumor efficiency and/or the lowest side effect) for the nano-based delivery system. However, it is completely different to translate a small-scale formulation into a large-scale production to satisfy industrial demands [67]. To overcome this issue, microfluidics technology and particle replication in non-wetting template (PRINT) technology can control chemical composition, drug loading, and surface properties of nanoparticles with precision [67,68,69,70,71].

#### 4.1.2. Toxicity and Side Effects of Nano-Based Medicines

Another major challenge associated with the translation of nano-based medicines to clinical practice is the nano–bio interactions which may cause toxicity and severe side effects [72]. The potential toxicity of nano-based medicines is usually caused by interactions with biological material which can generate immunoreaction, inflammation, or related disorders in the human beings. The toxic effect is greatly dependent upon various parameters such as size, zeta potential, and solubility of the formulations [73]. When nanoparticles or liposomes enter a biological system, they interact with and are absorbed by proteins [74]. This adsorption of protein onto the surface of nanoparticles or liposomes results in altering their size, surface charge, stability, dispersibility, pharmacokinetics, biodistribution, and toxicity profile [75,76]. Furthermore, it has been reported that many nano-based medicines have caused severe acute adverse immune reactions in vivo [77]. Szebeni et al. reported that liposomes were known to activate the complement (C) system, which could lead, in vivo, to a hypersensitivity syndrome called C activation-related pseudoallergy (CARPA) which has received increased attention as a safety risk of i.v. therapy with liposomes [78]. Furthermore, long circulation properties may also result in non-specific accumulation in the skin causing serious side effects such as hand-foot syndrome, or palmar-plantar erythrodysesthesia, as reported for PEGylated liposomal doxorubicin [79,80]. In addition, there are off-target risks, due to some of the antibody nanoparticles ligands that are also targets in normal cells [81]. Additionally, nanoparticles may deactivate immune responses and can be cleared by immune cells which become a further burden for the immune system and lead to even worsen the blood cancer [82]. Therefore, it is quite critical to investigate the physicochemical characteristics of nano-based medicines with respect to pathophysiology and heterogeneity of human diseases.

#### 4.1.3. Circulation and Clearance 

The liver and spleen are the two major organs of nano-based medicine uptake and clearance, as well as kidney, lung, and bone marrow that are also involved in this process [83,84]. Macrophages play a critical role during the clearance of nanoparticles or liposomes in these organs [85,86]. To maintain a long circulation profile and to decrease recognition by host cells, surface coatings have been developed to increase circulation times in blood [87,88]. Polyethylene glycol (PEG) and polyglycerol (PG) are highly hydrophilic molecules which can reduce the protein absorption and affect the composition of the proteins absorbed on the surface of nanoparticles. It is one of the commonly used materials for coating in nanoparticle preparation which can evade immune cells [89,90,91]. It has been reported that anti-PEG antibodies identified in patients could accelerate clearance of PEG-modified nanomedicines. They may also increase the rate of adverse events, such as allergic reactions [92]. Depletion of phagocytes is another strategy for enhancing the circulation time of nano-based medicines by using clodronate-loaded liposomes [93,94,95,96]. Furthermore, macrophage depletion by clodronate liposomes supports prevention of nanoparticle clearance from the peripheral blood and is also a tool to study the role of macrophages and other phagocytes in health and disease [97,98,99]. In addition, clodronate is a first-generation bisphosphonate that has been approved for the prevention and treatment of osteoporosis [100,101] and it has also shown potential therapeutic efficacy for treating chronic lymphocytic leukemia [102,103,104].

#### 4.1.4. Translational Study in the In Vivo Model 

The first step of a preclinical study to evaluate novel pharmaceuticals including nano-based medicines is in vitro testing in order to identify the biocompatibility and efficacy and it is usually performed on cancer cell lines. Some novel in vitro culture systems have been established recently to simulate the microenvironment of tumors such as 3D culture systems or organoid culture systems which allow evaluation of therapeutics in a microenvironment that is somewhat closer to the actual disease situation [105,106]. Although these novel in vitro systems can mimic the microenvironment of a tumor or the cell–cell interactions, an in vivo animal model is still required for the development of circulation, biodistribution, safety and efficacy profiles before the clinical trial process [107]. There are lots of factors that should be considered to improve in vivo animal models to study drug delivery and therapeutic efficacy in blood cancers. First of all, the cell line in the in vitro culture has been clonal selected for lots of passages which may have already changed the original profile of the disease. Therefore, using a primary tumor derived animal model rather than a cell line-based model should be considered in in vivo animal models. Secondly, different patient-derived models may response completely different to the same nanoparticles or liposomes. The difference in the sensitivity may also provide personalized precise medicine according to the patient’s personal genomic profile which could be further investigated. Last but not least, it is realistic that tumor growth in animal models is still different than in humans. There are many new medicines that have shown promising antitumor activities in animal models but are still less effective in clinical trials [108]. One more thing specifically applicable in blood cancer studies is that the immunodeficient strains are widely used to allow xenograft models to grow in animals. The lack of an immune system may alter the pharmacokinetics and the pharmacodynamics profile in vivo, especially for blood cancers that often involve the immune system [109].

#### 4.1.5. Interfere with the Bone Marrow for Blood Cancer Drug Delivery Systems 

Unlike solid tumors, blood cancers are usually the malignance in bone marrow. Nano-based medicines overcome a series of barriers to deliver an antitumor agent into the bone marrow or the tumor site. In addition, targeting malignancies inside the bone marrow is still a complex issue since it involves cancer stem/progenitor cell existence as well as bone marrow microenvironment induced resistances [110,111]. Therefore, disruption of the interactions between tumor cells and the bone marrow microenvironment via two or three different agents/drugs may achieve greater clinical responses to increase sensitization and overcome the resistance. However, the stable co-encapsulation of multiple agents into a single nano-based targeted drug delivery system might change the pharmacokinetic or pharmacodynamic profiles of combined drugs to achieve the additive or synergistic effect for tumor therapy for blood cancers [112,113]. In addition, the accumulation of antitumor drug inside the bone marrow could enhance the antitumor efficacy but also lead to cumulative toxicity to the normal hematopoiesis cells [114]. Therefore, the development of elaborate bone marrow-targeted systems is essential for specific delivery of antitumor drugs to tumor cells and to minimize the capture by healthy bone marrow cells in the bone marrow.

### 4.2. Non-Biological Challenges

#### 4.2.1. Commercialized Challenges

One of the major commercialized challenges associated with the clinical translation of nano-based medicines is the difficulty in formulating a controllable and reproducible synthesis process. Working in a laboratory on a small-scale formulation is much easier and highly dependent on the operator’s experience, which are not suitable for reproducible large-scale production. In addition, the formulation must be stable to allow long term storage and shipment which also makes the situation more complex [115]. It has been noticed that nanoplatforms with laborious and complex manufacturing processes rarely find their way into clinical practice due to the inconvenience caused to the pharmaceutical industrial.

#### 4.2.2. Policy/Regulation Challenges

Another urgent and major challenge that needs to be addressed is the huge gap between scientific research and the regulatory authorities. In most of the countries, the authorization of a new medicine is monitored by the government according to a series of policies and laws regarding safety profiles, industrial manufacturing practices, intellectual property protections, quality controls, etc. In the United States, the approval process for nano-based medicines is essentially the same as that for other drugs or biological medicines which are regulated by the Food and Drug Administration [116,117,118]. Unless there is specific consideration for a particular nano-based medicine, the development of nano-based medicines follows the typical drug-development process [119]. This regulation of guidelines for nano-based medicines has been questioned [120]. Timely and effective translation to market is highly affected due to the deficiency of clear regulatory and safety guidelines [121]. Currently, commercially available nano-based medicines on the market have passed the general regulatory standards for approval. However, these standards may not be sufficient and need further revision to confirm quality, safety, and efficacy for human use [76,114]. Nowadays, the rapid development of nanotechnology has contributed to its potential use in nanomedicine. There is an urgent need for more integrated regulatory policies which should be done by country governments. The new guidelines should benefit nanomedicine for patients by addressing any concerns delaying the releasing process for nano-based medicines [121].

## 5. Summary

Targeted delivery of therapeutic agents plays a pivotal role in the effective and safe treatment of blood cancers. Targeting B cell malignancies inside the bone marrow is still a biological issue due to cancer stem/initiating cell existence and bone marrow microenvironment-induced resistances. It is also notable that the accumulation of therapeutic agents inside the bone marrow or lymph nodes might also lead to cumulative toxicity to normal hematopoiesis stem cells or inhibit the immune response. Further investigations should focus on the specific delivery of therapeutic agents to tumor cells and on minimizing the capture by healthy cells in the bone marrow or lymph node. Recently, several new nanomedicines and drug delivery formulations have been successfully developed and clinically approved for treatment of many types of cancers, which indicates that effective and safe targeted formulations are expected to benefit the treatment of patients in the future. Scientists, industry bodies, and governments should work together to overcome the biological and non-biological challenges to make the translational research smoother for patients.

## Figures and Tables

**Table 1:** An overview of different types of blood cancers. Due to the scope of this review the somewhat rarer forms are not discussed here. Data were adopted from Cancer Statistics 2022 (USA), adapted with permission from ref. [1]. Copyright 2022 John Wiley & sons.

Types of Blood Cancers	Origin	New Cases	Deaths
		Est. in 2022	Est. in 2022
Multiple myeloma (MM)	B cells (plasma cells)	34,470	12,640
Acute myeloid leukaemia (AML)	Myeloid lineage hematopoietic precursors	20,050	11,540
B cell lymphoma (BL)	B cells and lymphocytes	80,910	21,170

## Sample Availability

Samples of the compounds are not available from the authors.
